# Increased Water Viscosity Enhances Water Intake and Reduces Risk of Calcium Oxalate Stone Formation in Cats

**DOI:** 10.3390/ani11072110

**Published:** 2021-07-15

**Authors:** Jean A. Hall, Melissa A. Vanchina, Blair Ogleby, Dennis E. Jewell

**Affiliations:** 1 Department of Biomedical Sciences, Carlson College of Veterinary Medicine, Oregon State University, Corvallis, OR 97333-4802, USA; 2 Pet Nutrition Center, Hill’s Pet Nutrition, Topeka, KS 66617-1587, USA; melissa_vanchina@hillspet.com (M.A.V.); blair_ogleby@hillspet.com (B.O.); 3 Department of Grain Science and Industry, Kansas State University, Manhattan, KS 66506, USA; djewell@ksu.edu

**Keywords:** feline, urine calcium oxalate titrimetric test, urine relative supersaturation for struvite crystals, urine stone formation, viscous water

## Abstract

**Simple Summary:**

Increasing water intake and decreasing urine concentration are recommended for cats that form stony concretions in the bladder or urinary tract. The purpose of this study is to determine if water with increased viscosity results in increased water intake in cats by taking advantage of the unique anatomy of the cat’s tongue. Cats delicately dip their tongue into water, pull their tongues back up to their mouths, and capture the water that follows. Lapping occurs by fluid adhesion to the dorsal part of the tongue’s tip and by lifting a liquid column through the tongue’s upward motion before jaw closure. Cats have the ability to balance gravity and water surface tension to ingest the maximum amount of water per lap. This observation led us to question whether changing the properties of water in a way that allows cats to lift more water per lap is possible. Increasing the viscosity of water was accomplished with 1% methylcellulose, an ingredient that is palatable to cats. Cats consuming 1% methylcellulose water had increased water intake (25% and 21% higher at 28 and 56 days, respectively), and increased water intake reduces the risk for calcium oxalate stone formation. The benefit of giving cats viscous water is that they can increase water consumption without having to change their natural drinking behavior.

**Abstract:**

The purpose of this study is to determine if water with increased viscosity results in increased water intake, thus lowering the risk of urolithiasis in cats. Twelve healthy adult cats were fed pre-trial standard dry maintenance food for 1 week and then randomized into two groups for the study phase. The cats continued to receive the same food but were provided either control (deionized) water or viscous (1% methylcellulose) water for two months and then switched to the other water type for two months in a cross-over study design with repeated measures. Complete blood counts, serum chemistry profiles, and urinalysis were performed at the initiation of the study and again at 1, 2, 3, and 4 months. Daily water consumption and energy intake for each cat were recorded. Body weights were assessed weekly. Cats consuming 1% methylcellulose water with increased viscosity had increased water intake (*p* < 0.001; 25% and 21% higher at 28 and 56 days, respectively). Increased consumption of water resulted in lower urine specific gravity (*p* = 0.04), serum creatinine (*p* = 0.02), and blood urea nitrogen (*p* = 0.002) concentrations (without changing serum albumin, glucose, and calcium concentrations or serum osmolality) and decreased urine calcium concentration (*p* = 0.01) compared with cats consuming control water. In addition, the increased water intake increased (*p* = 0.05) resistance to oxalate crystal formation.

## 1. Introduction

Cats produce very concentrated urine, which increases their risk for water deprivation and kidney health problems [1]. Increasing water intake could potentially help reduce the risk of developing dehydration following stressors such as anesthesia [2] and dental procedures [3]. Increased water intake and decreased urine concentration are recommended for cats with urolithiasis and idiopathic cystitis, as reviewed by Lulich and Osborne [4]. In 2018, urinary issues were the number one reason that cat owners took their cat to the veterinarian [5]. 

The daily water requirements of cats (mL/d) is roughly 1.2× resting energy requirements [6] or 1.5 to 2 mL of water per gram of dry matter (DM) intake [7]. Daily water intake (mL) has also been reported per kg of body weight (23 ± 10 mL/kg) [8] and per kcal of metabolizable energy (ME) ingested (0.63–0.71 mL/kcal ME) [9]; the latter is in agreement with what other researchers have reported: 0.6 to 0.7 mL/kcal ME for dry food [10,11] and 0.9 mL/kcal ME for wet food [12]. 

Researchers have employed several strategies, some unsuccessfully, to increase water intake in cats, including increasing food moisture through the use of wet food [13], increasing dietary protein [14], increasing dietary salt intake [15,16], and varying the water source [8,17]. Nutrient-enriched water has also been provided to improve measures of hydration in healthy cats [9].

Another strategy that we report here involves the use of viscous water to take advantage of the unique anatomy of the cat’s tongue. Cats’ tongues are covered with hundreds of sharp, scoop-shaped, and backward-facing keratin spines called filiform papillae [18]. However, only the tip of the tongue is used for lapping fluid. The tip is free of filiform papillae. Cats delicately dip their tongue into the water, pull their tongues back up to their mouths, and capture the water that follows. Lapping occurs by fluid adhesion to the dorsal part of the tongue’s tip and by lifting a liquid column through the tongue’s upward motion before jaw closure [19]. Cats have the ability to balance gravity and water surface tension to ingest the maximum amount of water per lap [19,20]. This observation led us to question whether changing the properties of water in a way that allows cats to lift more water per lap is possible. This was ultimately accomplished by increasing the viscosity of water with an ingredient that is palatable to cats. The objective of this study is to evaluate whether water with increased viscosity results in increased water intake in cats, thus lowering the calculated risk of urine stone formation.

## 2. Materials and Methods

All study protocols and this study were reviewed and approved by the Institutional Animal Care and Use Committee, Hill’s Pet Nutrition, Inc., Topeka, KS, USA (Permit Number: CP607), and have complied with the National Institutes of Health Guide for the Care and Use of Laboratory Animals [21]. Cats were individually housed for the majority of the day (>20 h/day) in spacious indoor rooms with natural light that varied with seasonal changes. Here, cats consumed food (ad libitum of a controlled amount to minimize weight gain) and water (ad libitum) and had their litter boxes in their individual condominiums. In addition, all cats were provided with regular opportunities for socialization and environmental enrichment by caretakers allowing the cats to mingle twice a day. The cats experienced behavioral enrichment through interactions with each other, daily interaction and playtime with caretakers, and daily opportunities to exercise on enclosed sun porches. Indoor room temperatures were kept constant, although cats would have been subject to ambient temperatures when outside sunning on porches. The cats were owned by the commercial funders of this research or their affiliates, who gave permission for them to be included in this study. At the conclusion of the study, all cats were returned to the Hill’s Pet Nutrition, Inc. colony.

### 2.1. Animals and Study Design

Twelve healthy adult cats were fed complete and balanced food that met AAFCO standards for maintenance food (dry cat food; Hill’s Pet Nutrition, Inc., Topeka, KS, USA) for 1 week and then randomized into two groups of six each for the study. During the study phase, the cats consumed the same food as the pre-trial food and were provided either control deionized water (Lindyspring, Topeka, KS, USA) or 1% methylcellulose water for two months and then switched to the other water type for two months. 

Methocel E4m food-grade modified cellulose (Dow Chemical, Midland, MI, USA) was used to prepare the methylcellulose water. Using a high shear mixer, 1% (weight for weight) of methylcellulose was blended into deionized water. During the shearing process, the product was placed under non-atmospheric, vacuum conditions. The viscous water was then processed using ultra-high temperature conditions (heating to ~140 °C, with a hold time of up to 4 s). The product was then cooled by flash cooling in a vacuum-sealed vessel and aseptically filled into shelf-stable packages to maintain all relevant product characteristics (i.e., viscosity and aroma). The measured viscosity of 1% methylcellulose was 282 centipoises, whereas the measured viscosity of deionized water was 4.9 centipoises.

In a pilot study, a 15 by 60 mm emery board was inserted into solutions of different methylcellulose concentrations, ranging from zero to 1% (1% being the methylcellulose concentration used in this study), and the amount of water retained on the emery board probe was measured by weight. We assessed whether the amount of water retained on the board was related to the concentration and, thus, the viscosity of the methylcellulose solution.

Cats in the first group were mean age 5.7 y (range 3–8 y); 3 males, 3 females; with initial body weight (BW) of 4.93 ± 1.42 kg (mean ± SD). Cats in the second group were mean age 6.5 y (range 5–8 y); 2 males, 4 females; with initial BW of 4.46 ± 1.02 kg (mean ± SD). All cats were domestic shorthair, spayed or neutered, and healthy, as determined by the colony veterinarian. In order to be included, cats had to have a normal physical examination, with normal blood taurine, thyroid hormone (T4), complete blood count, and serum chemistry profiles within the previous 12 months prior to the initiation of the study, and no evidence of concurrent disease. Cats were excluded if they had acute or chronic disease, abnormal physical examination or laboratory findings, or positive urine culture. Cats were removed from the study if they lost more than 10% of their BW, if their body condition score fell below two, if they refused to eat for three days, if they developed any medical conditions or adverse events that needed medication that conflicted with the study design (e.g., diuretics), or if they did not urinate during the collection period. 

Complete blood counts, serum chemistry profiles, urinalyses, and urine tests for stone risk were performed at baseline and again at 1, 2, 3, and 4 months. Thus, each cat had laboratory analyses performed after consuming the different water types for 28 and 56 days. Daily water consumption and food intake by each cat were recorded. Body weights were assessed weekly. Energy intake was expressed as kcals/BW_kg_^0.75^ as data from this cat colony showed that energy intake scaled best to the 0.75 power rather than the 0.67 power; the latter has been used in some studies to express the metabolic weight of cats. This is likely because cats from Hill’s colony are less obese than the average cat, and the smaller exponent considers the increased body weight associated with adiposity.

Once a week, an additional bowl was placed within the room, out of reach of the cats, to measure the evaporation rate for that day. The amount of evaporation from that bowl was subtracted from each cat’s calculated intake. If a cat played in or otherwise disturbed their water bowl during the intake measurement, that data point for the day was removed from the mean calculation.

Urine was collected at each assessment time (at days 27, 55, 83, and 111 of the study) over two days (48 h). The cats were trained to micturate in a litter box containing polypropylene beads. Urine was collected in thymol, which was used to inhibit microbial growth. Urine analysis (pH, urine specific gravity (USG), and dipstick analysis (IDEXX UA* Strips, IDEXX Laboratories, Inc., Westbrook, ME, USA)) as well as urine sediment analysis (semi-quantitative) and calculation of the urine protein:creatinine (UPC) ratio were performed. A refractometer (Reichert VET360, Reichert, Inc., Depew, NY, USA) was used to measure USG. The concentration of urine creatinine functioned as an internal reference. Both urine and serum creatinine used the same assay. The same assay was also used to measure urine and serum calcium concentrations. The benzethonium chloride turbidometric method was used to measure urine protein concentrations in urine supernatant. Calculated UPC ratios were determined as reported previously [22] and expressed as mg/dL protein:mg/dL creatinine.

An analysis of urine for relative super supersaturation (RSS) for struvite crystals was performed at the same time using the EQUIL 2 program [23,24,25]. In brief, a urine supersaturation ratio (unitless) is calculated by a computer program with respect to the common kidney urolith components. The EQUIL 2 program provides an evaluation of the state of urinary saturation based on pH and total concentrations (M/L) of specific analytes. We measured calcium, sodium, chloride, potassium, magnesium, ammonium, phosphate, citrate, sulfate, and oxalate concentrations. Thermodynamic stability constants are used to calculate free ion activities for urine ions. These free ion activities can then be used for calculating the supersaturation ratio of urine compared with what would form crystals in pure water. This test provides a theoretical risk for struvite stone formation.

In order to evaluate the calcium oxalate stone risk of each cat, a test called the calcium oxalate titration (COT) test was performed based on procedures adapted from Laube et al. [26,27,28], which we previously reported on for assessing theoretical resistance to oxalate crystal formation in cats [29]. In brief, the [Ca^+2^]/(added Ox^−2^) ratio is calculated per liter. In humans, this is also called the Bonn-Risk Index. A higher ratio indicates a greater risk of calcium oxalate crystallization, whereas a lower ratio suggests the urine has less risk of forming calcium oxalate crystals. The ratio is derived from the concentration of ionized calcium and the amount of oxalate that must be added to initiate crystal formation. 

### 2.2. Statistical Analysis

This was a cross-over study design with repeated measures. We chose no wash-out period because of the long treatment period before the first measurement, which was taken at 28 days. Statistical analyses for body weight, water intake, energy intake, serum analytes, and urinalysis parameters were performed using a repeated measures-in-time analysis of variance model in PROC MIXED in Statistical Analysis Software version 9.4 (SAS Institute, Cary, NC, USA). Fixed effects in the model were water type (control or viscous water), time (28 and 56 days), order (first control water or first viscous water; this term evaluates a carry-over effect), water type by order interaction, and water type by time interaction. Animal was the experimental unit and was included in the model as a random variable. If there was no carry-over effect, the order term and the interaction term for order were removed from the model. Differences between treatments were then evaluated through the main effects of water type, time, and water type by time interaction. All data are reported as least square means ± pooled standard errors of means (SEM). Significance was accepted as *p* ≤ 0.05, whereas *p* ≤ 0.10 was considered a trend. A COT test analysis was completed on natural logarithm (ln) values as the data were not normally distributed. All other reported data were normally distributed.

To investigate the independent variables affecting struvite RSS and oxalate crystal formation, we used a regression model (PROC CORR) with animal as the experimental unit. We used PROC GLM in SAS to estimate the best fit linear model for previously published predictors [29]. The interaction term between significant independent variables was used to calculate a linear function, and the correlations between this term and RSS or oxalate crystal formation were then determined.

The effects of treatment on the incidence of calcium oxalate and struvite crystals in urine were determined by comparing the number of cats with vs. without crystals across specific treatments using Fisher’s exact test. 

## 3. Results

One cat was removed from the study on day 97 because of low energy intake and BW loss. Missing data at the 4-month time point were handled by maximum likelihood using PROC MIXED [30]. 

Body weight tended to be affected by water type, in that body weight was higher in cats consuming viscous water (*p* = 0.06; Table 1). The initial body weight of all cats was 4.69 ± 0.36 kg. The range of body weights for cats consuming viscous water was 3.58 to 7.48 kg. For cats consuming control water, body weights ranged from 3.47 to 7.06 kg.

**Figure 1:**
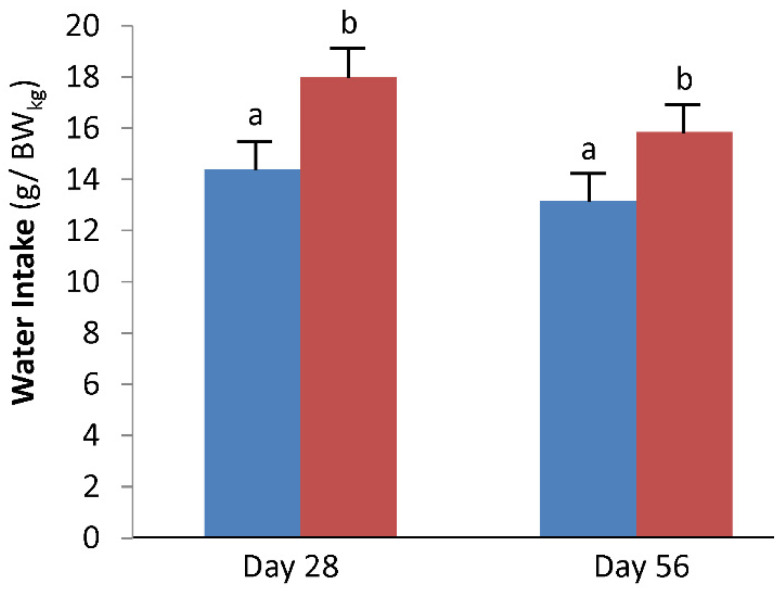
Water intake (g/BW_kg_ per day) after 28 and 56 days of treatment. Cats consumed either control water (deionized; blue; *n* = 6) or 1% methylcellulose water (viscous; red; *n* = 6) for 56 days and were then switched to the other water type for 56 days. Shown are least-square means ± pooled standard errors of means for all cats (*n* = 12) while consuming the control or viscous water type. ^a,b^ Means with different superscripts within a period are different. Water intake was significantly greater in both groups of cats at day 28 compared with day 56.

There was increased water consumption in cats drinking viscous water (expressed either as g/100 kcals per day or g/BW_kg_ per day; both *p* = 0.001; Table 1; Figure 1). In 10 of 12 cats, the mean daily water intake was greater for 1% methylcellulose water compared with deionized water. Using the means of intake per kg body weight, cats consumed 25.0% and 20.6%, (28 and 56 days, respectively) more water when consuming viscous water compared with control water. The range of water intake, expressed in g/day, for cats consuming viscous water was 39 to 144 g/d. For cats consuming control water, water intake varied from 25 to 106 g/d. There was also a significant effect of time on water intake (*p* = 0.04 for water intake expressed as g/100 kcals per day or g/BW_kg_ per day), in that water intake went down in cats consuming both types of water across time.

Energy intake was not affected by water type (*p* = 0.88; Table 1). The cats ingested, on average, 1.6 g water per gram of dry food, although as food intake increased, water intake increased at a rate of 2.2 times (slope of water intake vs. food intake = 2.2; r^2^ = 0.43). Therefore, as individual cats had higher food intake, there was a greater increase in water consumption.

All significantly different serum chemistries, as well as other selected serum chemistries, are shown in Table 1. While consuming viscous water, cats had decreased serum creatinine, BUN, cholesterol, and triglyceride concentrations compared with cats drinking deionized water (all *p* < 0.05). Serum albumin, glucose, and calcium concentrations were not different between treatment groups based on the type of water consumed. There was a significant effect of time on serum cholesterol and albumin concentrations (both *p* < 0.05). Cholesterol concentration increased in all cats across time, whereas albumin concentrations decreased in all cats across time. There was a significant water type by time interaction (*p* = 0.05) for triglyceride, in that cats consuming viscous water had decreased concentrations and cats consuming deionized water had increased concentrations across time.

Serum osmolality was calculated from serum chemistries using the following equation: 2(Na^+^ + K^+^) + glucose/18 + BUN/3 = serum osmolality (mOsm/kg). Serum osmolality was not affected by water type, but there was an effect of time (*p* = 0.03), in that osmolality increased from 4 to 8 weeks in cats consuming both control water and viscous water.

Regarding urinalysis parameters, there was no significant effect of water type on urine pH (Table 1), although there was a trend (*p* = 0.07) for the water type by time interaction. Cats consuming viscous water tended to have an increase in pH across time from initial values (6.36 ± 0.07), whereas cats consuming deionized water tended to have an increase in pH in the first 4 weeks, followed by a decrease in pH in the second 4 weeks. The range of urine pH in cats consuming viscous water for 56 days was 5.7 to 7.1, whereas the range in cats consuming control water for 56 days was 5.9 to 7.3.

There was a significant effect of water type on USG (*p* = 0.04; Table 1). The range of USG in cats consuming viscous water for 56 days was 1.026 to 1.068, whereas the range in cats consuming control water for 56 days was 1.032 to 1.078.

There was a trend of a significant effect of water type on the UPC ratio (*p* = 0.08; Table 1). The UPC tended to be lower in cats consuming viscous water. The range of UPC ratios for cats consuming viscous water for 56 days was 0.05 to 0.13; for cats consuming control water for 56 days, the range was 0.06 to 0.16.

There was a significant effect of water type on urine calcium concentration (*p* = 0.01; Table 1). Using the mean values of cats consuming both water types, there were 24% and 11% reductions (28 and 56 days, respectively) in urine calcium concentration in cats consuming viscous water compared with cats consuming control water. The range of calcium concentrations in cats consuming viscous water for 56 days was 1.76 to 11.6 mg/dL, whereas the range in cats consuming control water for 56 days was 2.75 to 18.1 mg/dL.

As kidney function is difficult to interpret with regards to urine calcium concentration without reference to urine creatinine concentration, fractional excretion of calcium (%) was calculated as urine calcium concentration/serum calcium concentration divided by urine creatinine concentration/serum creatinine concentration × 100. There was a trend of a significant effect of water type on the fractional excretion of calcium (*p* = 0.06; Table 1). Fractional excretion of calcium tended to be lower in cats consuming viscous water. The range of fractional excretion values in cats consuming control water for 56 days was 0.098% to 1.246%, whereas the range in cats consuming viscous water for 56 days was 0.094% to 0.412%. 

Struvite RSS values were similar in cats after consuming both water types (Table 1). The range of values in cats consuming control water for 56 days was 0.53 to 24.27, whereas the range in cats consuming viscous water for 56 days was 0.29 to 23.78. We used a second statistical model to investigate the independent variables affecting struvite RSS. In our previous paper [29], we observed that struvite RSS was predicted by USG and urine pH. In this study, the best fit linear model generated unique predicted values for struvite RSS in these cats. These predicted values had an *r-*value of the correlation of predicted vs. measured struvite RSS of 0.89 (*p* < 0.001). 

There was a significant effect of water type on COT test values (*p* = 0.05; Table 1). Cats consuming viscous water showed enhanced titratability to added oxalate before forming calcium oxalate crystals. We used the natural log (ln) transformation of the COT values because the original values were not normally distributed. The range of values in cats consuming control water for 56 days was 1.9 to 6.4 ln 1/L, whereas the range in cats consuming viscous water for 56 days was −0.1 to 4.6 ln 1/L. Using the mean values of cats consuming both water types, there were 30% reductions (at days 28 and 56) in COT test values in cats consuming viscous water compared with cats consuming control water. Thus, consuming viscous water decreased the risk of oxalate crystal formation. 

We used a second statistical model to investigate the independent variables affecting oxalate crystal formation. In our previous paper [29], we observed that the risk of calcium oxalate crystal formation (estimated from the COT test) was predicted by USG and the fractional excretion of calcium. In this study, the best fit linear model generated unique predicted values for the risk of calcium oxalate crystal formation in these cats. These predicted values had an *r-*value of the correlation of predicted vs. measured COT test values of 0.94 (*p* < 0.001).

Urine sediment analysis revealed that the incidence of calcium oxalate crystals tended to be higher in cats while consuming control water (*p* = 0.10). Specifically, three cats had calcium oxalate crystals, all while consuming control water. Two cats had no crystals while consuming viscous water but developed crystals after consuming control water for 4 weeks; subsequently, they were negative at 8 weeks. One cat had crystals after consuming control water for 8 weeks, but none were observed after transitioning to viscous water. No calcium oxalate crystals were observed in cats after consuming viscous water for 27 and 55 days.

On the other hand, struvite (magnesium, ammonium, phosphate) crystals were noted in cats after consuming control water or viscous water. Four cats had struvite crystals after consuming control water for 4 weeks, including a cat that had calcium oxalate crystals after consuming control water for 4 weeks; none were noted at 8 weeks. Three cats had struvite crystals after consuming viscous water (three after 4 weeks and two persisting at 8 weeks). 

In the pilot study, whereby we assessed whether the amount of water retained on the emery board was related to the concentration and, thus, viscosity of the methylcellulose solutions, we found that the methylcellulose concentration was linearly correlated to the amount of water retained on the emery board (*r* = 0.99; *p* < 0.0001).

## 4. Discussion

Fundamentally, cats consuming 1% methylcellulose water with increased viscosity had significantly increased water intake compared with cats consuming control deionized water. This increased consumption of water resulted in changes typically associated with increased glomerular filtration (i.e., lower USG and lower serum BUN and creatinine concentrations) without changing serum albumin and glucose concentrations or serum osmolality. Additionally, there was no change in serum calcium concentrations associated with increased water intake. Water intake in cats receiving both control water and viscous water decreased over time, from 28 to 56 days, but remained higher in cats consuming viscous water. Correspondingly, calculated serum osmolality increased in cats consuming both water types over time, from 28 to 56 days. We do not know the cause for the main effect of time on water intake. There were no observed changes in behavior and no environmental changes. It is possible that the novelty of having a different type of water changed drinking behavior (drank less) over time.

In cats, water intake can be affected by food moisture [6]. If fed a commercial dry food, cats will drink about 1.5 to 2 mL of water for each gram of dry food consumed [31]. This proportion, a 2:1 ratio of water to dry matter, is similar to that of animals that are typical prey for carnivores [32] and represents approximately 0.5 mL water/kcal ME intake [7]. In our study, cats consuming viscous water ingested, on average, 1.6 g of water for each gram of dry food consumed or 0.43 g/kcal ME. Thus, water consumption, although increased in cats consuming viscous water compared with cats consuming control water, was not in the range of overconsumption. Overconsumption is extremely rare in healthy cats but can be induced if they are offered a free choice of water after prolonged dehydration [6]. Evaporation losses can also falsely increase water consumption data, but evaporation was carefully controlled for in our study.

Normally, osmoreceptors in the hypothalamus sense changes in body fluid osmolality and send signals to the antidiuretic (ADH) synthesizing/secreting cells in the supraoptic and paraventricular nuclei of the hypothalamus. In addition to affecting the secretion of ADH, changes in plasma osmolality lead to alterations in the perception of thirst. An increase in plasma osmolality of 2% to 3% produces a strong desire to drink. As water consumption in cats drinking viscous water increased without affecting serum osmolality, it is assumed that ADH secretion was decreased and, thus, water permeability of the collecting ducts in the kidney also decreased. Although ADH concentrations in plasma were not measured, a lower USG in cats consuming viscous water supports this conclusion.

Energy intake was similar in cats consuming viscous water compared with cats consuming deionized water and similar to what is recommended for overweight cats consuming dry commercial food [33]. There was a trend for higher body weight in cats consuming viscous water. Although the increase was minor (0.8% higher at the end of treatment), we cannot rule out changes in physical activity as an explanation for increased body weight while consuming viscous water. 

The higher fiber intake associated with consumption of viscous water was the most likely explanation for decreased serum cholesterol and triglyceride concentrations while consuming viscous water. A meta-analysis of 67 controlled trials in human nutrition quantified the cholesterol-lowering effect of major dietary fibers and showed that various soluble fibers reduced total cholesterol by similar amounts [34]. Methylcellulose is a non-fermentable, 100% soluble fiber known to slow the rate of digestion of dietary fats [35]. It has been shown in cats that the addition of various fiber sources to foods reduces energy and nutrient digestibility, whereas the effects on postprandial serum cholesterol and triglyceride concentrations are dependent on the fiber source [36].

Increased water intake had the benefit of reducing the risk of calcium oxalate stone formation (reduced COT values), which is associated with the observational finding of no calcium oxalate crystals in the urine of cats while consuming viscous water. We have previously [29] concluded that the COT test is superior to measuring other variables in urine and calculating a CaOx RSS because the COT test takes into account the inherent characteristics of urine to resist CaOx stone formation. Lower COT test numbers theoretically indicate a reduced risk of stone formation, which is measured as an increased capacity of urine to resist crystal formation in the presence of added oxalate. The changes that allowed for a reduced risk of calcium oxalate stone formation were a reduced urine calcium concentration and a tendency for decreased fractional excretion of calcium. Fractional excretion is the fraction of blood calcium that is excreted in the urine, which tended to be lower in cats consuming viscous water. The risk of oxalate crystal formation (determined by the COT assay) was also predictable using fractional excretion of calcium and USG measurements. Cats consuming viscous water had decreased USG and tended to have decreased fractional excretion of calcium compared with cats consuming control water, both of which decreased the risk of calcium oxalate crystal formation. In light of our previous paper [29], it is not surprising that specific urine analytes were good predictors of stone risk in these cats as well. Specifically, the COT test is highly related to USG and the fractional excretion of calcium [29]. As increased water intake changed these urine analytes, COT values were also reduced. 

We did not see a benefit for reducing struvite RSS with viscous water consumption and observed struvite crystals in the urine of cats while consuming viscous water; however, the reduction in USG with viscous water consumption is likely a benefit for preventing stone formation. From our previous study [29], struvite RSS is highly related to USG and urine pH; urine pH was not altered by increased water intake in this study.

We have previously shown that feeding cats increased dietary long-chain polyunsaturated fatty acids (PUFA) can theoretically lower the risk of urine stone formation by decreasing USG and urine calcium concentration; water intake (approximately 72 g/d) was unchanged [29]. It is possible that the effects on urolith prevention might be greater when combining both increased dietary PUFA concentrations and offering viscous water. In the previous study, we suggested that altered prostaglandin E_2_ production in the kidney may have lowered urine calcium excretion [37]. Other proposed mechanisms to decrease urine calcium concentration besides dilution (increased water intake) and drugs (e.g., thiazide diuretics) include high-fiber diets, which have been shown to reduce intestinal absorption and urinary excretion of calcium in humans [38,39,40], although oxalate concentration in urine increased with bran fibers. It is possible that methylcellulose acts in a similar way to these fibers; even though serum calcium concentrations were not changed, reduced intestinal absorption may be associated with reduced urinary excretion. 

The benefit of giving cats viscous water is that they can increase water consumption without having to change natural drinking behaviors and preferences. In the pilot study, whereby we attempted to mimic the unique papillous anatomy of the cat’s tongue using an emery board probe, we showed that increasing the viscosity of the methylcellulose solution was linearly correlated to the amount of water retained on the emery board. This suggests that increasing the viscosity of water with methylcellulose allows greater lift per dip. Our results, i.e., increased water consumption in cats while consuming viscous water, can be explained by the emery board study. Future studies to monitor drinking frequency and number of laps per cat per day are needed to confirm increased lift per dip in cats, as we observed with emery boards.

Increasing water intake and decreasing urine concentration are recommended for cats with urolithiasis and idiopathic cystitis, as summarized by Grant [8]. In addition, increased water intake in cats could potentially help lower the risk of dehydration following well-health procedures such as anesthesia [2] and dentistry. For example, cats with impaired renal function, e.g., increased serum concentrations of symmetric dimethylarginine (SDMA) and creatinine, noted prior to dentistry, may benefit from increased water intake before and following recovery from the dental procedure. Providing viscous water as a preventative healthcare measure increased the water intake of healthy cats in this study.

A potential limitation of the study was the lack of baseline water intake data. We considered control (deionized) water intake to be representative of baseline water intake, but this could be different from potable water intake. In a pilot study using healthy cats from the same colony, whereby potable water intake was measured (unpublished results), potable water intake was about 10% lower than the deionized water intake reported in this study. Thus, it is feasible that viscous water intake would be even higher compared with potable water intake. We also did not have a wash-out period between the two water treatments, which some studies might consider a limitation, although there was a long period (28 days) before the first measurements were made after the cross-over. This is likely longer than any wash-out period would have been. In addition, the cross-over study design used each cat as its own control, so any variation associated with an individual cat was handled by including order in the model. Future studies to investigate the effects of food ingredients (e.g., PUFA content) coupled with viscous water intake would be interesting.

## 5. Conclusions

The main finding of this study is that cats consuming 1% methylcellulose (viscous) water have increased water consumption compared with cats consuming deionized water. This is associated with decreased serum creatinine and BUN concentrations, decreased USG, and decreased urine calcium concentration. In addition, increased water intake has the benefit of reducing the risk of calcium oxalate stone formation (reduced COT values).

## Figures and Tables

**Table 1:** Body weight, water intake, energy intake, concentrations of serum analytes, and urinalysis parameters (mean ± SEM) of cats at baseline (initial) and after consuming control (deionized) water or 1% methylcellulose (viscous) water for 28 and 56 days.

	Control Water	Viscous Water	*p*-Values		
Number of Cats, *n*	12	12	Water Main Effect	Time Main Effect	Water by Time Main Effect
Body Weight (kg)					
28 days	4.63 ± 0.31	4.73 ± 0.31	0.06	0.84	0.17
56 days	4.68 ± 0.31	4.70 ± 0.31			
^£^ Water Intake (g/100 kcals)					
28 days	35.8 ± 3.57	45.8 ± 3.57	0.001	0.04	0.60
56 days	32.5 ± 3.57	40.2 ± 3.57			
Water Intake (g/BW_kg_)					
28 days	14.4 ± 1.27	18.0 ± 1.27	0.001	0.04	0.56
56 days	13.1 ± 1.23	15.8 ± 1.23			
^£^ Energy Intake (kcals/BW_kg_^0.75^)					
28 days	59.3 ± 2.7	58.6 ± 2.7	0.88	0.79	0.41
56 days	58.0 ± 2.7	59.4 ± 2.7			
Calculated serum osmolality (mOsm/kg) ^‡^			0.98	0.03	0.75
28 days	320.9 ± 1.0	320.5 ± 1.0			
56 days	322.7 ± 1.0	323.0 ± 1.0			
Serum concentrations:					
^£^ Creatinine (0.97–1.97 mg/dL) ^§^					
28 days	1.36 ± 0.06	1.28 ± 0.06	0.02	0.54	0.95
56 days	1.34 ± 0.06	1.26 ± 0.06			
Urea (14.9–29.5 mg/dL) ^§^					
28 days	19.6 ± 0.8	17.8 ± 0.9	0.002	0.40	0.93
56 days	20.1 ± 0.8	18.2 ± 0.9			
Cholesterol (107–300 mg/dL) ^§^					
28 days	204.4 ± 9.3	192.2 ± 9.3	0.002	0.04	0.91
56 days	213.0 ± 9.3	199.9 ± 9.3			
Triglycerides (20–183 mg/dL) ^§^					
28 days	22.3 ± 1.7	21.9 ± 1.7	0.03	0.59	0.05
56 days	25.3 ± 1.7	20.2 ± 1.7			
^£^ Albumin (2.7–3.8 mg/dL) ^§^					
28 days	3.3 ± 0.08	3.2 ± 0.08	0.49	<0.001	0.84
56 days	3.1 ± 0.09	3.1 ± 0.09			
Glucose (65–115 mg/dL) ^§^					
28 days	84.6 ± 3.4	85.6 ± 3.4	0.33	0.42	0.18
56 days	90.5 ± 3.5	84.1 ± 3.5			
^£^ Calcium (8.9–10.9 mg/dL) ^§^					
28 days	9.44 ± 0.14	9.39 ± 0.14	0.92	0.61	0.43
56 days	9.43 ± 0.14	9.46 ± 0.14			
Urinalysis parameters:					
pH					
28 days	6.29 ± 0.10	6.14 ± 0.10	0.76	0.75	0.07
56 days	6.18 ± 0.11	6.29 ± 0.11			
Specific gravity					
28 days	1.053 ± 0.003	1.046 ± 0.003	0.04	0.99	0.33
56 days	1.051 ± 0.003	1.048 ± 0.003			
Protein:creatinine ratio					
28 days	0.108 ± 0.007	0.092 ± 0.007	0.08	0.51	0.71
56 days	0.111 ± 0.008	0.100 ± 0.008			
Calcium (mg/dL)					
28 days	6.29 ± 0.99	4.75 ± 0. 99	0.01	0.88	0.30
56 days	5.92 ± 1.01	5.25 ± 1.00			
^£^ Fractional excretion of calcium (%)					
28 days	0.266 ± 0.062	0.192 ± 0.066	0.06	0.45	0.84
56 days	0.306 ± 0.062	0.215 ± 0.064			
Struvite relative supersaturation (unitless)					
28 days	6.22 ± 1.8	5.03 ± 1.8	0.39	0.89	0.11
56 days	3.90 ± 1.9	7.75 ± 1.9			
^£^ Calcium oxalate titration test * (ln 1/L)					
28 days	3.59 ± 0.34	3.24 ± 0.34	0.05	0.87	0.99
56 days	3.57 ± 0.36	3.22 ± 0.35			

^£^ There was a significant water type by order interaction, so order and water type by order interaction were left in the statistical model. ^‡^ Serum osmolality was calculated from the following equation: 2(Na^+^ + K^+^) + Glucose/18 + BUN/3. ^§^ Normal reference interval for the laboratory. * Calcium oxalate titration test analysis was completed and reported on natural logarithm (ln) values as the data were not normally distributed.

## Data Availability

The data presented in this study are available within the article.
